# Trimeric HIV Env provides epitope occlusion mediated by hypervariable loops

**DOI:** 10.1038/srep07025

**Published:** 2014-11-14

**Authors:** Carlos G. Moscoso, Li Xing, Jinwen Hui, Jeffrey Hu, Mohammad Baikoghli Kalkhoran, Onur M. Yenigun, Yide Sun, Lassi Paavolainen, Loïc Martin, Anders Vahlne, Carlo Zambonelli, Susan W. Barnett, Indresh K. Srivastava, R. Holland Cheng

**Affiliations:** 1 Department of Molecular and Cellular Biology, University of California, Davis, CA 95616; 2 Novartis Vaccines and Diagnostics Inc., 45 Sydney Street, Cambridge, MA 02139; 3 Department of Biological and Environmental Science/Nanoscience Center, University of Jyväskylä, FI-40351 Jyväskylä, Finland; 4 Commissariat à l'énergie atomique et aux énergies alternatives, Institut de Biologie et Technologies de Saclay, Service d'Ingénierie Moléculaire des Protéines, Gif-sur-Yvette F-91191, France; 5 Karolinska Institutet, Structural Virology, Clinical Microbiology/University Hospital, 171 77 Stockholm, Sweden

## Abstract

Hypervariable loops of HIV-1 Env protein gp120 are speculated to play roles in the conformational transition of Env to the receptor binding-induced metastable state. Structural analysis of full-length Env-based immunogens, containing the entire V2 loop, displayed tighter association between gp120 subunits, resulting in a smaller trimeric diameter than constructs lacking V2. A prominent basal quaternary location of V2 and V3′ that challenges previous reports would facilitate gp41-independent gp120-gp120 interactions and suggests a quaternary mechanism of epitope occlusion facilitated by hypervariable loops. Deletion of V2 resulted in dramatic exposure of basal, membrane-proximal gp41 epitopes, consistent with its predicted basal location. The structural features of HIV-1 Env characterized here provide grounds for a paradigm shift in loop exposure and epitope occlusion, while providing substantive rationale for epitope display required for elicitation of broadly neutralizing antibodies, as well as substantiating previous pertinent literature disregarded in recent reports.

Membrane fusion between HIV and host cells is mediated by the Env complex on the surface of the viral membrane envelope^1^. The Env complex is comprised of three copies each of gp120 and gp41, arranged as a trimer on the viral membrane^2^. The capacity of Env-based trimeric immunogens to elicit a broad and potent immune response could provide a significant degree of protection against viral infection. Given the propensity for distinct antibodies to preferentially target the trimeric form as opposed to monomeric gp120, there is an impetus to characterize with fidelity the quaternary structure of Env-based immunogens so as to pinpoint targets for rational immunogen design.

Previously, we showed that the structure of gp140ΔV2TV1^3^ including a 30-residue truncation in the second hypervariable (V2) loop^4^,^5^,^6^ had a concave apex and a depressed trimeric center, a view supported by recent cryotomography and cryoelectron microscopy (cryoEM) work^7^,^8^,^9^. Recently, an alternative architecture of the Env complex has been put forth^10^,^11^,^12^, with a large cap at the trimeric apex that houses the V2 and V3 hypervariable loops. Such an arrangement does not take into consideration the myriad studies suggesting close association between gp120 and gp41^13^,^14^,^15^,^16^,^17^,^18^,^19^; instead, these recent publications suggest that there is only minimal gp120-gp41 interaction, limited to the N- and C-termini. Further, the recent publication of the structure of a clade A strain BG505 SOSIP gp140 trimer in complex with the Fab of PGV04^20^, accompanied by a crystal structure of the same gp140 in complex with PGT122 Fab^21^ agree with the apical cap view of trimer arrangement. However, another recent cryoEM structure of a clade A strain KNH1144 SOSIP trimer in complex with the Fab of the 17b antibody again shows a marked lack of trimeric “cap” apex and thus the lack of cavity. A more recent article from the same group^22^ indeed claims that the presence of a large cavity in the center of the trimer is likely an artifact due to limited resolution, and indeed shows a convex apex with no cap in the native, “closed” quaternary state.

Our current results challenge the prevailing perspective, and posit that instead of being situated at the trimeric apex, the V2 loop is situated at the basal region of the trimer, oriented toward the adjacent gp120 subunit and associating with that subunit's V3 loop, forming the quaternary neutralizing epitope (QNE). Indeed, exactly how juxtaposed V2 and V3 loops from the same subunit, a tertiary epitope, would form the basis for the QNE, preferentially recognized on trimeric Env^8^,^23^,^24^ is difficult to ascertain. Further, other corroborating data, including increased exposure of gp41 upon V2 deletion^25^,^26^, increased V3′ exposure following V2 deletion^6^,^27^,^28^, and increased exposure of V4^29^,^30^,^31^, all support our model of apical V4 exposure and basal V2 location, with interprotomeric contacts between V2 and the adjacent V3′. Our model also paints an indirect role for V2 in CD4 binding site (CD4BS) occlusion, as observed in several reports^6^,^32^,^33^,^34^,^35^,^36^; by engaging the adjacent subunit, the interprotomeric contacts decrease the trimeric diameter, and thus decrease apical accessibility to the CD4BS, in contrast to the current view of direct steric blockage of the CD4BS by a solid cap containing hypervariable loops.

Biochemical and structural characterization of the recombinant, soluble trimeric immunogen gp140^3^,^5^,^34^,^37^ has yielded results demonstrating that deletion of the V2 loop enhances binding of CD4BS-targeting antibodies, and slightly increases CD4 binding, suggesting that V2 may be involved in occlusion of the CD4BS. The V2 loop can acquire length, a finding correlated with enhanced viral escape from host immune responses^38^. Our recent structure of a clade C gp140 immunogen with a partial, 30-residue V2 deletion suggests that the quaternary location of the V2 loop is proximal to the viral membrane, and oriented toward the adjacent counterclockwise gp120 subunit^3^. The V2 loop has also been implicated in formation of a quaternary epitope formed by V2 and V3′ that is preferentially recognized in Env trimers by broadly neutralizing antibodies (bNAbs) PG9 and PG16^24^ and 2909^39^, which have recently been structurally characterized^8^,^40^. However, the formation of such an epitope in the quaternary context remains to be investigated.

Attempts at elucidating the structure of the Env complex to understand its mechanism of action have yielded disparate results with no clear consensus^8^,^41^,^42^,^43^,^44^,^45^. Earlier tomograms of membrane-embedded Env trimers suggested that the V2 loop was located at a region proximal to the viral membrane at the base of the trimeric spike^41^,^42^, as was also proposed in trimeric modeling of the SIV gp120 monomeric X-ray structure^46^. More recent tomography structures of membrane-embedded trimers have suggested that the location of the V2 loop is at the trimer apex “cap”^8^,^43^,^45^. A more striking example of this apical “cap” followed in the description of Env spikes extracted from detergent-treated virions^10^,^12^. Structures of clade A and B gp140 immunogens dispelled at least in part the notion of a closed cap at the trimer apex, but showed little to almost no density associating the three gp120 subunits in one structure^7^. More recent SIV spike cryo-tomograms from the same group^47^ revert back to the “cap” over the threefold axis, while also claiming an “open” state. Such structural interpretations of an apical V2 quaternary location are inconsistent with studies using biochemical^16^,^18^ and scanning mutagenesis methods^14^,^15^,^17^,^19^, and as such, the location of V2 remains unresolved.

Here we present the structures of a clade C trimeric, full-length soluble gp140 immunogen from the TV1 strain in the absence and presence of the CD4-mimetic miniprotein CD4m^48^, and compare these structures to our previous study of immunogens with a partial V2 truncation^3^. The quaternary arrangement hints at a mechanism for epitope occlusion that may offer pertinent insight into gp41-independent gp120-gp120 interactions.

## Results

### Full-length gp140 reconstructions with and without CD4m

**Figure 1:**
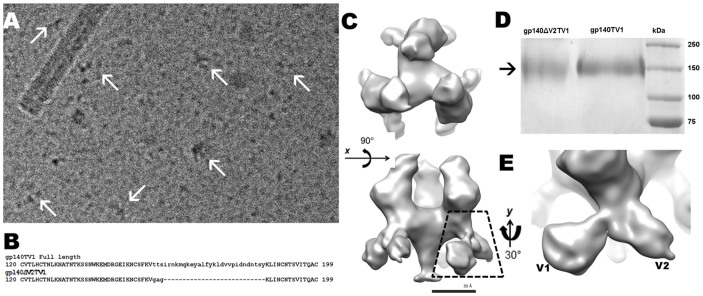
Structure of full length gp140. (A) Raw cryoelectron micrograph of gp140. Arrows point to particles embedded in vitreous ice. (B) Sequence alignment of gp140 and gp140ΔV2, showing 30-residue deletion near tip of V2^34^. (C) Top and side views of gp140 exhibit a clockwise handedness and protrusions from the basal trimer regions. Resolution is ~21 Å at 0.5 FSC. (D) SDS-PAGE of gp140 and gp140ΔV2, with molecular weights at approximately 140 kDa. (E) Detail in (C) bottom panel, accentuating protruding densities at base of gp140 assigned to V1 and V2 loops.

Isosurface rendering of the gp140TV1 (hereinafter gp140) density map showed that the trimer structure retained the propeller-shaped motif that gp140ΔV2 exhibited (Figs. 1A, 1C). The three subunits of gp120 displayed clockwise handedness, a pointed gp41 hub, and dimensions of 100 Å in diameter and 125 Å in height, taller than that of the gp140ΔV2 trimer (Figs. 1C). While retaining a congruent overall morphology, there are a few key differences between the constructs, aside from the slight difference in molecular weight (Fig. 1B, 1D). The gp140ΔV2 density map was approximately 90 Å in height, while the gp140 density map is about 125 Å in height. Another important difference is the smaller degree of tilt away from the threefold *z* axis; the gp140ΔV2 construct shows gp120 subunits at a tilt of approximately 25°, whereas the gp140 construct exhibits a tilt of about 15°–20°. A more relevant feature is the presence of a tail-like density at the putative location of the V2 loop (Fig. 1C, 1E). Two distinct densities at the location of the V2 loop can be seen, pointing in opposite directions, and likely displaying the branched, adjacent V1/V2 loops. Additionally, the center trimeric region was also similar to that of gp140ΔV2, suggesting that this region anchors the three subunits to the central stalk. If each gp120 subunit is assigned a long axis through the molecule at 90°, 210° and 330° (S_1_, S_2_ and S_3_, respectively), all normal to the threefold *z* axis, then each subunit would appear tilted away from the *z* axis about the S*_x_* axis by ~25°, similar to the unliganded gp140ΔV2 structure. The three gp120 regions appear to be more closely associated near the trimer apex, with density at the outermost tip of each gp120 subunit oriented toward the threefold axis. The wedge-shaped trimer arm region is quite consistent when compared to the gp140ΔV2 map.

### Presence of V1/V2 density proximal to viral membrane

Comparative analysis of both maps places the location of V2 on the outer edge of each trimer fan blade proximal to the viral membrane, supporting our earlier docking of coordinates in the gp140ΔV2 density map (Fig. 1E). The V1 loop faces the adjacent clockwise subunit, whereas the V2 loop would be in a position proximal to the adjacent counterclockwise subunit, likely mediating gp41-independent gp120-gp120 interactions. The V1/V2 loops appear as a branched pair of densities facing in opposite directions. The density attributed to V1 is ~38 Å in length, and the density corresponding to V2 is about 45 Å. These dimensions correspond well with the length expected from the number of residues in each loop.

### Immunogold labeling of gp140 reveals basal V2 and V3′ locations

**Figure 2:**
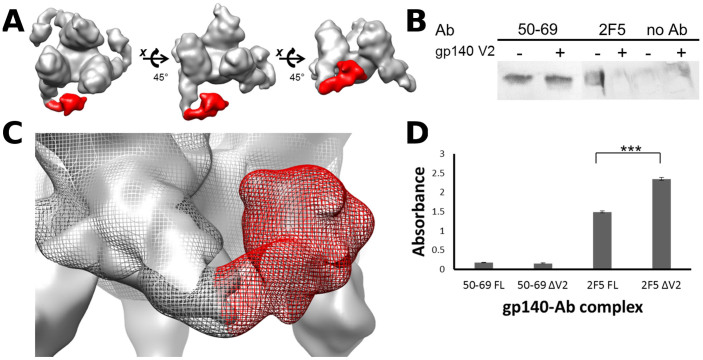
Structure of gp140-PG16 complex reveals basal V2 location, and deletion of V2 results in exposure of basal gp41 epitopes as detected by western blot and ELISA. (A) The gp140 trimer was incubated with PG16 Fab and imaged by cryoEM. Structure of the gp140-PG16 complex reveals a basal density emanating from the putative location of the V2 loop (red). Resolution is ~26 Å at 0.5 FSC. (B) Superimposition of the gp140 unliganded map (solid) and the gp140-PG16 complex (mesh) reveals that binding of PG16 occurs at the basal V2 density (red mesh). (C) Western blot of gp140 and gp140ΔV2 constructs labeled with antibodies 50–69 and 2F5, targeting apical and basal gp41 epitopes respectively, shows that exposure of the 50–69 epitope (targeting the intrahelical disulfide-containing loop on gp41) is unaffected by V2 deletion, while 2F5 epitope exposure (targeting the gp41 membrane-proximal external region) is greatly enhanced after V2 deletion. Blots shown are representative results. (D) ELISA of gp140 and gp140ΔV2 bound to 50–69 and 2F5 similarly reveal that while 50–69 epitope exposure is unaffected by V2 deletion, 2F5 epitope exposure is significantly enhanced by V2 deletion (*n* = 3).

Conjugation of gold-labeled PG16 Fab′ fragments with trimeric gp140 followed by single particle reconstruction of gp140-PG16 complexes revealed a density protruding from the basal V2 location (Fig. 2A). The gp140-PG16 map confirmed the basal location of V2, as well as the interprotomeric location of the QNE. Superimposition of the gp140 unliganded map with the gp140-PG16 map confirmed the basal protruding density apportioned to V2, as well as additional density assigned to the PG16 Fab (Fig. 2B).

### Accessibility of gp41 epitopes upon V2 deletion by western blot and ELISA

In order to determine the relative accessibility of apical or basal gp41 epitopes as a function of V2 deletion, we probed full-length and ΔV2 gp140 constructs with anti-gp41 antibodies targeting apical (50–69, targeting the intrahelical disulfide-containing loop) and basal (2F5, targeting the membrane-proximal external region) epitopes by western blot. There were no differences in 50–69 epitope accessibility between full-length and truncated gp140, while there was a marked difference in accessibility to 2F5 epitope, with full-length gp140 shielding the epitope and gp140ΔV2 displaying increased access to it (Fig. 2C). Epitope access between constructs was also determined utilizing the same antibodies by ELISA (Fig. 2D), with similar results.

### Comparison of gp140 maps before and after CD4m conjugation

**Figure 3:**
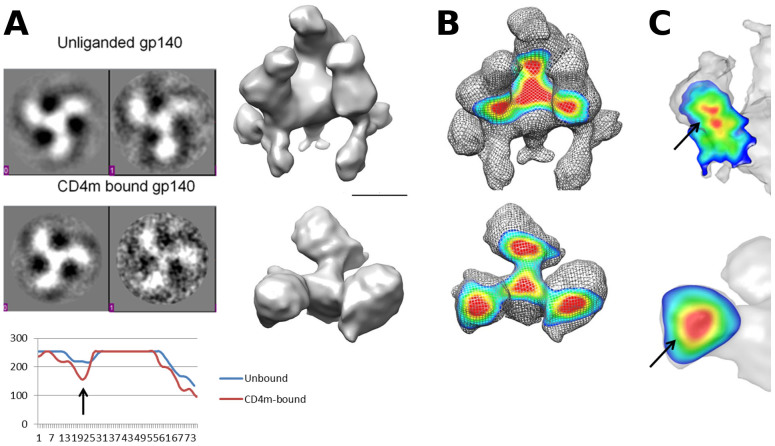
Comparison of gp120-gp41 contacts and inner-outer domain separation in full length gp140 and gp140ΔV2. (A) 2D analysis of gp140 and gp140-CD4m class averages by cursor profile reveals distinct weakening of gp120-gp41 interface region, as exemplified by graph at bottom (arrow). The cursor profiles were measured from the center of the trimer outwards to the edge of the circular mask. Oblique views of unbound gp140 (top) and CD4m-bound gp140 (bottom) reveal trimeric flattening and subunit rotations elicited by ligand binding. Scale bar = 50 nm. Resolution of gp140-CD4m structure is ~26 Å at 0.5 FSC. (B) Top: Density map of gp140 shows a strong interface between gp120 and gp41. Bottom: Density map of gp140-CD4m complex shows largely diminished gp120-gp41 interface. (C) Slice through gp140 structure (top) shows a density separation, likely representing the inner and outer gp120 domains, while slicing through gp140-CD4m (bottom) shows a collapse of domains, without discernible gap between inner and outer domain.

The gp140 map was conjugated with CD4m, and the morphology of the trimer in the CD4m-triggered state was quite consistent with our previous observations. The diameter of the triggered map remains at 110 Å, while the height is 80 Å, a marked reduction in height from the unliganded form resulting from subunit tilting away from the *z*-axis (Fig. 3A). There is a pronounced dissociation away from the threefold axis, and the interaction between each trimer arm and the trimeric center is quite weakened. Rotation of subunits about axes perpendicular from the *z*-axis result in a previously occluded face on each subunit being newly exposed for perpendicular access (Fig. 3A).

### Gp120-gp41 interface diminution, outward density shift and gp120 domain coalescence

We performed preliminary 2D analysis of gp140 bound to CD4m, and confirmed our earlier results of an outward interface diminution. By taking class averages showing top views of both unliganded and CD4m-liganded gp140 (Fig. 3A), and taking cursor profiles of class averages through the gp140 arms, a decrease in intensity in the CD4m-conjugated structure corresponding to the gp120-gp41 interface is apparent when compared to the unliganded gp140 map. The density diminution was confirmed by analyzing slices of the gp140 unliganded map and the gp140-CD4m map (Fig. 3B), with a clear gap in high intensity voxels at the gp120-gp41 interface in the CD4m-bound map.

A higher level of detail was gleaned from comparative analysis of the two density maps by slicing through the volume, with distinct subunit domains evident from density gravity centers (Fig. 3C). Previously, distinct and discrete density peaks present within the volume attributed to gp120 suggested a separation between domains on gp120. The current map of gp140 also shows a similar density separation when slicing through the volume; this finding corroborates the initial observations of domain separation described for the gp140ΔV2 density map (Fig. 3C). Also, the inner and outer domains of gp120 in the trimer arms were shown to coalesce following CD4 binding, agreeing with our earlier observation (Fig. 3C).

### Comparison of gp140 and gp140ΔV2 maps reveal a gradient of “open” states mediated by both V2 deletion and CD4m binding

**Figure 4:**
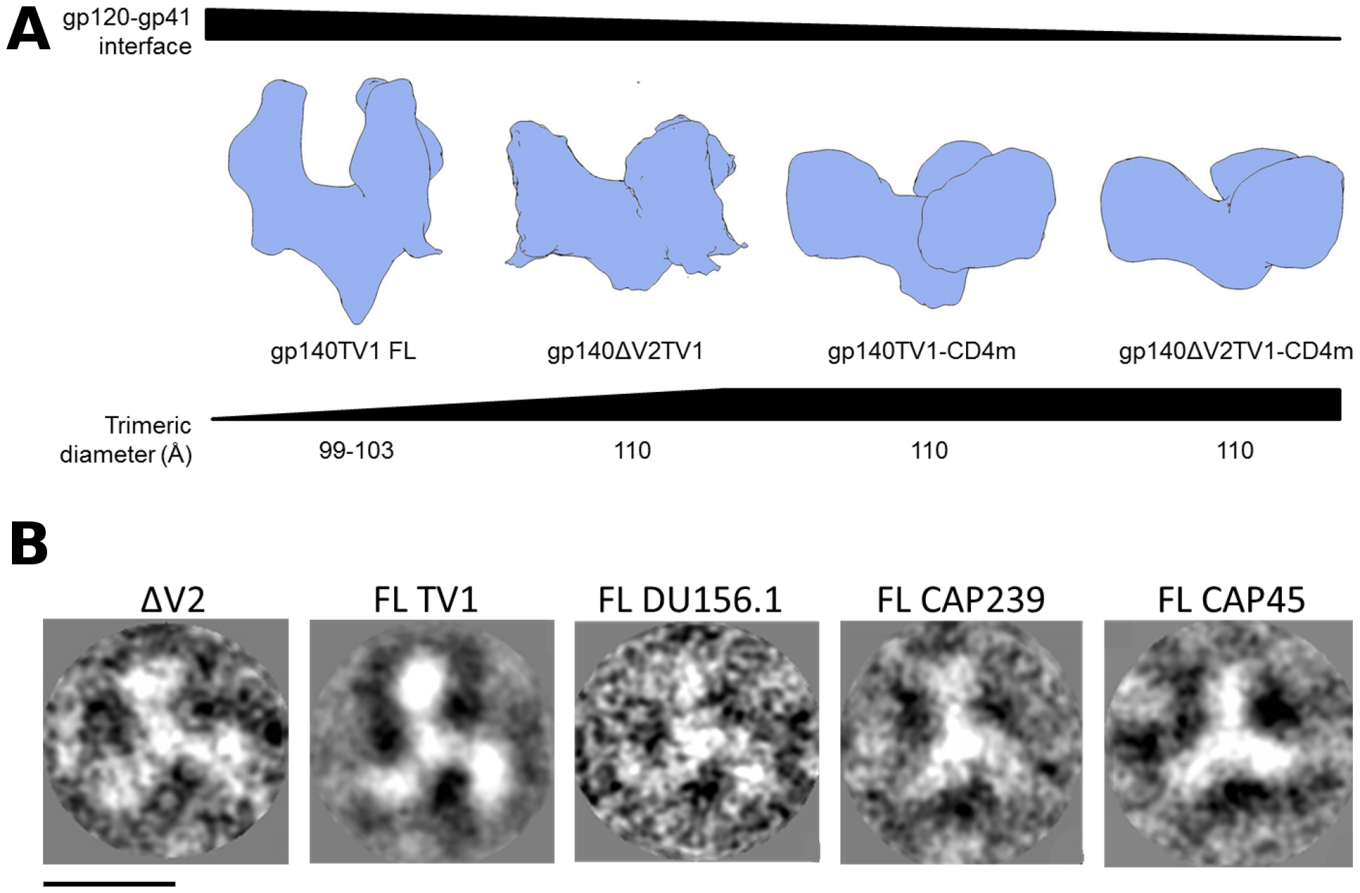
Schematic representation of gp140 demonstrates intersubunit associations, relaxation of trimeric diameter upon deletion of V2. (A) The gp140 trimer was compared with the gp140ΔV2, gp140-CD4m and gp140ΔV2-CD4m maps. The degree of gp120-gp41 interface strength appears to decrease as a function of both V2 deletion and CD4m conjugation, with the gp140ΔV2-CD4m map representing the most “open” state. Inclusion of the V2 loop also results in a smaller trimeric diameter (100 Å), though the diameter remains unchanged in the gp140ΔV2, gp140-CD4m and gp140ΔV2-CD4m maps (110 Å). (B) 2D comparison of gp140ΔV2TV1 with full length constructs gp140TV1, gp140DU156.1, gp140CAP239 and gp140CAP45 reveals that full length constructs all had smaller trimeric diameters (99–103 Å) than gp140ΔV2TV1 (110 Å), indicative of closer intersubunit associations in full-length constructs than in ΔV2 constructs. Scale bar = 75 Å.

The gp140 and gp140ΔV2 maps, with and without CD4m, were determined to have a gradient of gp120-gp41 interface “open” states, as determined by the extent of intensity at the interface region. The unliganded gp140 map had the strongest interface between gp120 and gp41 observed of all four maps, followed by the gp140ΔV2 map, then the gp140-CD4m and lastly the gp140ΔV2-CD4m map (Fig. 4A). As such, it appears that both the V2 loop and CD4m binding contribute to the degree of “open” state, putatively exposing novel epitopes at the gp120-gp41 interface region. Given the propensity of V2 to promote interprotomeric gp120-gp120 contacts via the QNE, we also observed a smaller trimeric diameter when comparing the gp140 and the gp140ΔV2 maps. The trimeric diameter remained unchanged between the gp140ΔV2, gp140-CD4m and gp140ΔV2-CD4m maps, resulting from the outward density shift promulgated by CD4m binding (Fig. 4A). Likewise, we observed that the smaller trimeric diameter is apparent in other clade C trimers, namely from the CAP45.2.00.G3, CAP239.2.00.G3J and Du156.1 strains (Fig. 4B). Measurements of trimeric diameter of ΔV2 compared to full-length strains revealed that the full length strains had smaller trimer diameters (99 to 103 Å) compared to ΔV2 (110 Å).

### Location of V3 & V4 loops, CD4 binding angles

**Figure 5:**
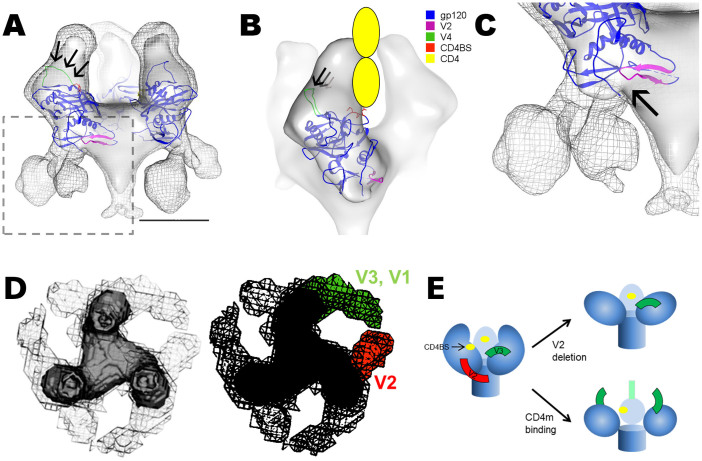
Docking of coordinates into gp140 and gp140-CD4m density maps and location of epitopes, and 2D segmentation of unbound gp140 map. (A) Side view of gp140 structure with SIV unliganded gp120 X-ray coordinates (PDB: 2BF1) docked. V1/V2 stem is in magenta, CD4 binding site is red, and V4 loop is green. Arrows point to potential N-linked glycosylation sites on V4 near the trimer apex. Solid surface represents predominant density, with weaker density shown in mesh. Scale bar = 50 Å. Dashed rectangle outlines detail shown in (C). (B) Likely binding orientation of CD4 (yellow) to gp140. Note the restricted accessibility to the CD4BS conferred by density near the threefold, attributed to heavy glycosylation in the V4 region. Arrows are shown in same orientation as in (A). (C) Juxtaposition of the truncated V1/V2 loop stem with the bifurcated density proximal to the viral membrane confirms our earlier V2 location. Arrow points to V1/V2 stem, as V1/V2 was truncated from the coordinates. (D) 2D segmentation of unbound gp140 map at low gradient magnitudes (mesh) and high gradient magnitudes (solid), from a top view, reveals that the variable loop regions at the base of the trimer disappear when map is visualized at high gradient intensities, suggesting that these regions are structurally malleable and not rigid. Core of the protein at the center is evident at high gradient magnitudes, suggesting that there is little change in voxel intensity rate of change defining the core of the structure, as opposed to the variable loops. At right, the proximity of the basal V2 (red) and apical V3 (green) promote intersubunit contacts, decreasing trimeric diameter in the unliganded state. (E) Model of quaternary effects of V2 deletion and CD4m binding. At left, the full length trimer shows a robust gp120-gp41 interface and interprotomer interactions between V2 and V3′ loops. The gp120-gp41 interface is partly diminished following V2 deletion, while interprotomer contacts are abrogated, resulting in a relaxed trimer with a larger diameter. Binding of CD4/CD4m results in further weakening of the gp120-gp41 interface, subunit rotation (as exemplified by the rotated CD4BS), and enhanced exposure of the V3 loop.

Coordinates of unliganded gp120 (PDB 2BF1, SIV) were docked into the density map, resulting in good agreement (correlation coefficient of 0.79) (Fig. 5A–C). The coordinates varied only slightly from the docking position in the gp140ΔV2 trimer. The location of the truncated V2 loop in the coordinates corresponds again to the visible densities emanating from this location. Coordinates of CD4m-bound gp120 (PDB 2I5Y, HIV-1) were docked into the gp140-CD4m map, again with good agreement (correlation coefficient of 0.77). Docked coordinates agreed well with our previous docking in the CD4m-bound state, with the CD4BS oriented facing the adjacent counter-clockwise subunit.

The V3 loop is situated on the inner face of the gp120 subunit trimer fan blade, facing the adjacent clockwise gp120 subunit. Adjusting the contour to ~0.75 σ above the mean intensity revealed a tail-like density protruding from the putative location and extending toward the neighboring clockwise gp120 subunit. The density attributed to this loop appears to be in close proximity to the V2 loop from the adjacent subunit (about 35 Å apart), close enough to ostensibly be accessible by one Fab fragment. Such a quaternary epitope would constitute increased intersubunit gp120-gp120 interactions when compared to the gp140ΔV2 construct, resulting in the decreased trimer radius. The V4 loop appears to be situated near the trimer apex (Fig. 5A–B), with its multiple asparagine residues likely contributing to increased electron density at this region. Additionally, the V1 loop appears to emanate from the same face of gp120 as the V3 loop, though from a location proximal to the viral membrane. The V1 and V3 loops are also about 40 Å apart, and the measured volumes of the V1, V2 and V3 loops at 0.75 σ are 20640 Å^3^, 15870 Å^3^, and 12250 Å^3^, respectively. The density map of gp140 reaffirms our previous findings suggesting that the CD4 binding loop (encompassing primarily residues 368–370) is perpendicularly exposed and thus available for receptor binding (Fig. 5B).

### 2D segmentation of unliganded and CD4m-liganded maps reveals structural malleability of variable loops

To determine the rigidity of variable loops, we computed 2D histograms based on gradient intensity of voxels, so as to visualize structurally stable core regions of the trimer as well as more structurally labile regions. Taking the derivate of intensities can augment detection of the solvent-protein interface, as well as differentiate between structurally rigid and labile regions. Taking regions from the 2D histogram, corresponding to a fixed contour at different gradient intensity values, revealed distinct density profiles not observed by traditional 1D volume histogram cutoffs. The gp140 unliganded, 2D-segmented map revealed that the protruding basal regions as well as the apical region were present at low gradient intensities but not at higher gradient intensities (Fig. 5D), revealing a core central density that remained at higher regions in the 2D histogram. The top view of the unliganded full-length gp140 map after 2D segmentation, at two different gradient intensities (Fig. 5D), revealed close proximity of variable loops at lower gradient intensity, indicative of close intersubunit interactions mediated by structurally labile hypervariable loops.

## Discussion

The location of the V2 loop presented herein, substantiating our previous work, suggests that V2 indirectly occludes the CD4BS through steric clashes of V4 glycans at the trimeric apex, near the three binding sites for CD4, through intersubunit association with the neighboring subunit's V3′ loop. Biochemical evidence of gp41 epitope accessibility upon V2 deletion corroborates our finding that while apical gp41 exposure is unaffected, V2 deletion results in exposure of basal gp41 epitopes targeted by mAb 2F5.

The juxtaposition of V2 and V3′ loops from different subunits explains why partial deletion of V2 confers enhanced CD4 binding capability^6^; by associating with each other, the V2 and V3′ loops mediate closer intersubunit interactions and decrease the trimeric diameter, reducing accessibility to the trimer apical regions including the CD4BS. Earlier studies describing altered binding properties of gp140 upon partial V2 deletion^5^,^6^,^33^,^34^ substantiate our quaternary location of the V2 loop in association to the enhanced exposure of neutralizing antibody (NAb) epitopes. Our current structure not only shows protruding density from the base of the gp120 subunits proximal to the viral membrane, populated by the V1/V2 loops (Fig. 1E), but also resolves the bifurcation between the V1 and V2 loops. Deletion of the V2 loop increases exposure of the gp41 membrane proximal external region (MPER)^25^,^26^, agreeing with our observed structural feature that V2 is proximal to the viral membrane and would sterically hinder access to the gp41 MPER.

The current density map, with the wedge-like gp120 subunit densities amenable to docking of unliganded coordinates, again corroborates our previous docking orientation, such that CD4 approaches the trimer at an angle of approximately 60°–90° from the horizontal plane (Fig. 5B). The exposed nature of the CD4BS would facilitate CD4 binding, while the heavily glycosylated face on V4, putatively at the trimeric apex, would guide the CD4 receptor toward the immunogenic and conserved CD4BS.

A recent report on Moloney murine leukemia virus, another retrovirus, indicates that Env undergoes subunit rearrangement following furin cleavage^49^. The finding that laterobasal protrusions are present in mature, cleaved forms of the virus, while apical densities are present in uncleaved, immature virus, bolster our findings that basal protrusions assigned to hypervariable loops are present in mature HIV-1 Env spikes.

The V3′ loop protrudes laterally toward the adjacent clockwise gp120 subunit and V2 loop, thus providing a footprint for QNE-recognizing antibodies such as PG16 and 2909^24^,^39^. The location of V3′ subsequent to CD4 binding renders it perpendicularly exposed, as previously suggested^3^,^43^. Such a gp120 subunit rotation about an independent axis would expose the cryptic CD4i epitope, and thereby could also abrogate intersubunit V2-V3′ contacts, contributing to the induced “open” conformation^3^.

The locations of V2 and V3′ on gp120 in the trimeric context appear to preclude the possibility of their joint accessibility on a single gp140 protomer, where the tips of V2 and V3 are found upwards of 60 Å apart, as observed from PDB coordinates 2B4C^50^. Introducing a PNGS to the V2 loop of SF162 gp140 (K160N) allows PG16 to bind with high affinity in either the monomeric or the trimeric forms^51^. The juxtaposition of V2 and V3, demonstrated here in the trimeric form, is much more feasible to provide the binding site for PG16 based on two adjoining protomers.

The V3 loop is occluded in the trimeric form of Env^52^, and proteolytic susceptibility is induced upon CD4 binding^53^. Moreover, deletion or mutation of V2 confers an increased immune response toward V3^54^. Such observations indicate that associations between V2 and V3′ result in V3′ occlusion in the unliganded trimer, as well as the subsequent quaternary rearrangements we previously reported^3^.

The location of V4 herein presents an alternative to current views that V2 dominates the apical trimer region. Glycosylated asparagines in V4 could provide the immunologically silent face^55^ necessary for immune evasion, and the location of these CD4BS-proximal glycans could provide a selective evolutionary advantage to HIV-1 virions presenting a recessed conserved primary epitope, as opposed to hypervariable loops comprising a cap over the trimer center. Although acquisition of length in the V2 loop has been correlated with neutralization resistance^38^, it is unlikely that a cap already formed at the trimer apex would gain neutralization resistance from increased length. A more likely scenario is that the degree of neutralization resistance observed in Env trimers with increased V2 length^38^ is conferred due to intersubunit gp120-gp120 interactions, with the longer V2 increasing contacts with the adjacent V3′ loop. In this scenario, V4 would be apically located, with the clade C gp140 construct including up to six PNGSs on and proximal to V4, which are represented by the increased density near the threefold axis (Fig. 5A–B). Given that V4 has been shown to elicit some of the earliest antibody responses^56^, its apical location corroborates previous observations that modifications of PNGSs in V4 are significant during HIV-1 adaptation to a novel host^57^.

A large site of immune evasion at the V4 and V5 locations has been identified^58^, which is heavily glycosylated and orients the immune response toward V3. Chen and colleagues point out that the bridging sheet forms aberrantly upon binding to b12, F105 and b13^58^, and that this bridging sheet abrogation may play a part in the potently (b12) and weakly (F105 and b13) neutralizing ability of these antibodies. Further, the observation that the V4 loop is involved in early autologous NAb response in HIV-1 subtype C-infected patients^56^ is an indicator that this region is likely highly exposed in the Env trimeric complex.

In contrast to earlier tomographic Env structures, recent structures of clade A and B gp140^7^ agree much better with the structural features of our current gp140 and previous gp140ΔV2^3^, in which the trimer apex does not contain a cap but rather displays concavity and clear separation between the gp120 subunits at the threefold axis. Similarly, Harris and colleagues recently drew on our earlier report of gp140ΔV2^3^ and reported evidence of a “closed” native conformation and an “open” triggered conformation. Their findings, though suggesting a solely quaternary event of trimeric conformational rearrangement, do not take into account the extensively reported gp120 tertiary conformational change and CD4-induced epitope presentation; instead, a rotation moving the V2 loop away from the threefold axis seems sufficient to delineate between the native, “closed” state and the CD4-triggered “open” state. Our structures reveal features that account for the conformational change in gp120 outlined by X-ray structures of gp120 in the unliganded state^46^ and in the CD4-bound state^59^. More recent examples of gp140 in the “closed” state that virtually abandon the heavy apical “cap” in favor of a concave apex, in line with our observations, have been recently reported^22^. Lastly, recent cryoEM and crystal structures of a clade A SOSIP trimer with a truncated MPER show elements of the apical cap, without the large central cavity^20^,^21^, though it is plausible that glycosylation at the apex may have confounded residue assignment in this region. Further differences between the gp140-PGT122 and gp140-PGV04 complexes and our native structure include the absence of a stalk-like gp41 region, which may be due to the MPER truncation present in the BG505.664 construct, and its full inclusion in the gp140TV1 construct. The position of V2 and V3 at the apex of the BG505.664 trimer structures does not deviate from our findings in the elicited, CD4-bound conformation, in which the V2 and V3 loops occupy a more apical location; our observations are that in the native, unliganded state, not shown in the BG505.664 structures, the V2 loop occupies a basal location that partially shields gp41.

## Conclusion

The ability of oligomeric gp140 to elicit NAbs with potency and breadth, outlining its feasibility as a vaccine candidate, has been a point of debate. Clade B gp140SF162 immunogens with a 30-residue deletion in the V2 loop^33^ have several strong components, namely a trimeric arrangement, ability to bind to potent NAbs and CD4i antibodies affinity approximating wild-type Env, glycosylation profiles closely mimicking wild-type Env, and similar CD4 binding capability, all pointing to gp140 as a viable vaccine candidate. The use of trimeric, recombinant soluble immunogens was partly validated by the observation that gp140ΔV2SF162 was capable of eliciting potent NAbs in a phase I clinical trial^60^, as well as by recent reports that boosting with trimeric gp140 proteins increased antibody titers in non-human primates^61^ and that trimeric gp140 elicits more potent and broad NAbs than monomeric gp120^23^. Recent reports of unliganded gp120 core constructs exhibiting the same conformation as CD4-bound gp120^62^ are recognized, which illustrate the importance of V2 and V3 in conformational modulation.

We present the structure of a full-length, clade C trimeric gp140 immunogen, and compared this structure to our previous structure of a clade C gp140 trimer with a partial V2 deletion^3^. A model was formulated through which V2 mediates gp41-independent gp120-gp120 contacts, decreasing the trimer diameter and occluding crucial gp120 epitopes. Additionally, we identified the juxtaposition of variable loops from different gp140 subunits, which provide the QNE that is preferentially targeted by antibodies such as PG16 and 2909^24^,^39^. Evident from our study, the V2 loop renders crucial conserved epitopes unexposed to the immune system by indirectly shielding the CD4BS and other epitopes, as well as by forming the QNE between the hypervariable loops from two adjacent subunits. Combined with the closely clustered glycosylation on the V4 loop at the trimer apex, the full-length V2-mediated Env conformation provides a significant degree of protection and evolutionary advantage to dispel attempts at elicitation of potent and broad NAbs.

## Methods

Methods to purify gp140 have been described elsewhere^34^. Briefly, Chinese hamster ovary cells expressed gp140 after transfection, and soluble gp140 trimers were purified using a *Galanthus nivalis* agarose lectin column, a diethylaminoethyl column, and a hydroxyapatite column, sequentially, in order to achieve high purity (>95%). Methods to image soluble gp140 trimers embedded in vitreous ice using cryoelectron microscopy have also been described previously^3^. Reconstruction, docking and interpretation of resultant gp140 density maps were done in the same manner^3^.

### Purification and sample preparation of gp140

The characterization and purification of subtype C, full-length oligomeric gp140 was performed in the same manner as gp140ΔV2^3^,^34^. Briefly, gp140 was expressed in Chinese hamster ovary cells and purified to homogeneity using a four step chromatography purification protocol. First, a *Galanthus nivalis* agarose (GNA) lectin column was used to capture the glycosylated Env construct, followed by a diethylaminoethyl (DEAE) column to capture acidic proteins and nucleic acids. A ceramic hydroxyapatite (CHAP) column was used to capture contaminating proteins while Env flowed through, and size exclusion chromatography isolated the trimeric form. Samples were prepared on holey carbon grids as previously described^3^,^37^, with a solution concentration of 0.1 mg/ml. Solutions of gp140 were diluted with Tris buffer (20 mM, pH 7.9) and 50 mM NaCl was added. Incubation of gp140 at 0.1 mg/ml with CD4m in excess concentration (10:1 molar ratio) was done overnight at 4°C. The CD4m miniprotein is a 27-residue CD4 mimicking peptide based on a scyllatoxin scaffold^48^, which has been shown to elicit a nearly identical gp120 conformation as CD4^63^. Additional gp140 strains CAP45.2.00.G3, CAP239.2.00.G3J and Du156.1 were prepared by similar methods and graciously provided by Jonathan Heeney (University of Cambridge, UK).

### Gold conjugation to Fab′ fragments and incubation with gp140

The antibody PG16, preferentially targeting V2 but also V3 to a lesser extent, was provided by the NIH AIDS Reagent Program. PG16 was digested using a Pierce Fab′ digestion kit (Thermo Fisher Scientific Inc., Rockford, IL) and conjugated to 1.4 nm gold fragments Nanogold® (Nanoprobes Inc., Yaphank, NY). PG16 was digested to F(ab′)_2_ fragments, reduced using 50 mM Tris(2-carboxyethyl) phosphine hydrochloride (TCEP, Sigma Aldrich, St. Louis, MO) for 10 min to yield reduced Fab′ fragments, and conjugated to gold particles for 30 mins, following manufacturer's protocol. The Fab′-gold conjugates were incubated with gp140 at a molar ratio of 3:1 at 4°C overnight.

### Cryoelectron microscopy and particle selection

Samples in vitrified ice were imaged using a JEOL 2100F field emission electron microscope at 200 kV, with an electron dose of approximately 15 e^−^/Å^2^. For the native state, 113 micrographs and 64 micrographs for the CD4m-bound state were recorded at 80,000× magnification (Fig. 1A) and used for single particle reconstruction with the EMAN software package^64^. Roughly 3300 individual native particle images were selected by a semi-automated particle selection protocol, as well as 4800 particles for the CD4m-bound structure. For the PG16-bound reconstruction, 2300 particles were selected and classified for reconstruction.

### Image processing, three-dimensional reconstruction and docking of X-ray coordinates

Phase contrast transfer function (CTF) correction was performed using *ctfit* in EMAN. The resultant particles were centered by autocorrelation, bandpass filtered, and submitted for refinement, as previously done^3^,^37^. The density map had previously published X-ray coordinates of SIV gp120^46^ docked into it using UCSF Chimera^65^ and Situs^66^. Improvement of resolution was achieved by systematic elimination of low-quality factor particles from refinement.

### Western blot and ELISA

Western blots were performed by incubating gp140 and gp140ΔV2 at 0.3 mg/ml with 50–69 and 2F5 antibodies (NIH AIDS Reagent Program) at 2 μg/ml, transferring to PVDF membrane overnight, blocking with 5% milk for 3 h, then washing in 0.1 M PBS-T 3X. The gp140-2F5, gp140ΔV2-2F5, gp140-50-69, and gp140ΔV2-50-69 complexes were then incubated in anti-human alkaline phosphatase-conjugated secondary antibody (Life Technologies, Grand Island, NY) diluted 1:500 for 1 h, washed in 0.1 M PBS-T, and the membranes were developed in SIGMAFAST™ BCIP®/NBT (Sigma-Aldrich, St. Louis, MO).

ELISAs were conducted on 96-well plates by adsorbing gp140 at 0.3 mg/ml, incubating with 50–69 and 2F5 at 2 μg/ml, applying anti-human alkaline phosphatase-conjugated secondary antibody (Life Technologies, Grand Island, NY) diluted 1:500, then adding PnPP substrate (Sigma-Aldrich, St. Louis, MO). Plate readings were done at 405 nm.

### 2D histogram segmentation by gradient intensity-based region growing

In order to separate densities with low gradient intensities, representing structurally labile regions, from more structurally rigid regions, we performed edge detection using gradient magnitude calculation and scatterplot region growing to generate a 2D histogram using BioImageXD (University of Jyväskylä, University of Turku, Finland). Regions were selected to differentiate from low and high gradient magnitudes.

## Author Contributions

C.G.M. and R.H.C. collected/analyzed data and wrote manuscript, L.X. collected/analyzed data, J.H.1, J.H.2, M.B.K. and L.P. analyzed data, O.M.Y. performed assays, Y.S. contributed reagents, L.M., A.V., C.Z., S.W.B. and I.K.S. provided fruitful discussion and analysis, and contributed reagents.
